# Enriched Environment Significantly Reduced Senile Plaques in a Transgenic Mice Model of Alzheimer’s Disease, Improving Memory

**DOI:** 10.3389/fnagi.2018.00288

**Published:** 2018-09-26

**Authors:** Janaina Balthazar, Natalia Mendes Schöwe, Gabriela Cabett Cipolli, Hudson Sousa Buck, Tania Araujo Viel

**Affiliations:** ^1^Department of Pharmacology, Institute of Biomedical Sciences, University of São Paulo, São Paulo, Brazil; ^2^School of Arts, Sciences and Humanities, University of São Paulo, São Paulo, Brazil; ^3^Department of Physiological Sciences, Faculdade de Ciências Médicas, Santa Casa de São Paulo School of Medical Sciences, São Paulo, Brazil

**Keywords:** Alzheimer’s disease, transgenic mice, spatial memory, enriched environment, brain resilience, senile plaques, amyloid-beta scavenger, SR-B1

## Abstract

Alzheimer’s disease (AD) is associated with a progressive dementia, and there is good evidence that it is more pronounced in individuals that have fewer stimuli during their lives. Environmental stimulation promotes morphological and functional changes in the brain, leading to amplification of cognitive functions, and has been described in humans and animals. In this study, we evaluated the effects of enriched environment (EE) stimulation on spatial memory and senile plaque formation in transgenic mice PDGFB-APPSwInd (TG) that overexpress the human amyloid precursor protein, normally resulting in an increased density of senile plaques. We compared this group of EE stimulated transgenic mice (TG-EE) with an EE stimulated control group of age-matched C57Bl/6 wild type animals (WT-EE). Both groups were exposed to EE stimulation between the ages of 8 and 12 months. As controls of the experiment, there were a group of TG mice not exposed to EE (TG-Ctrl) and a group of WT mice not exposed to EE (WT-Ctrl). The TG-EE group presented improved spatial memory when compared to the TG-Ctrl animals. In addition, the TG-EE group showed a 69.2% reduction in the total density of senile plaques in the hippocampus when compared to the TG-Ctrl group. In this group, the concentration of senile plaques was greater in the dorsal part of the hippocampus, which is linked to spatial localization, and the reduction of this density after the submission to EE was as high as 85.1%. EE stimulation had no effect on the density of amyloid-β (Aβ) oligomers. However, amyloid scavenger receptor class B member 1 (SR-B1) density was significantly decreased in the TG-Ctrl mice, but not in the TG-EE mice, suggesting that cognitive stimulation had an effect on the formation of a cognitive reserve that could prevent the accumulation of senile plaques. It is suggested that the stimulation of old mice by EE for 4 months led to the formation of brain resilience that protected the brain from the deposition of senile plaques, one of the hallmarks of AD, leading to improvement in spatial memory.

## Introduction

Alzheimer’s disease (AD) is a progressive and fatal neurodegenerative process and pharmacological therapy can only delay its progression or reduce some symptoms. Prominent pathological characteristics of the disease are the aberrant accumulation of amyloid plaques and the development of neurofibrillary tangles, which lead to cognitive impairment over the course of the development of the disease. There is some evidence, however, showing that soluble oligomers of amyloid-β (Aβ) peptides as well as the imbalance between the production and clearance of Aβ are strongly related to the synaptic dysfunction and cognitive deficits in the early stages of onset of the disease (Laganowsky et al., 2012; Figueiredo et al., 2013; Selkoe and Hardy, 2016). The clearance of Aβ to reduce the amyloid load is one of the strategies that has been proposed to promote a better quality of life for AD patients (Wyss-Coray et al., 2001; Bates et al., 2009; Xin et al., 2018). It is believed that increases in this load precede the onset of the disease by approximately 20 years. So, clearance rising can be a striking strategy for future pharmacological/non-pharmacological management of the disease. There has been an increase in the number of studies related to strategies to reduce the disease progress and improve quality of life, mainly related to cognitive aspects (Duara et al., 2009; Ghezzi et al., 2013; Cummings et al., 2017).

As it is well known, normal aging brings a series of alterations in the biological process. However, it is also known that changes in life course cause changes in aging configuration (Settersten, 2017). It means that a change someone does in lifestyle (doing or not physical exercise, for instance), the way someone faces health (positively or negatively) or even self-esteem can lead to a better or worse aging process (McEwen, 2007; Phillips et al., 2017). In this way, the brain is also a target of the life course strategies chosen by an individual and this can determine the construction of a cognitive reserve along life.

Cognitive stimulation is one of the strategies suggested to develop and maintain cognitive reserve and aid in the process of neuroprotection. The hypothesis of cognitive reserve was constructed from the observation that people with brain pathology had an apparently normal cognitive function (Barulli and Stern, 2013). In this way, education, mental training and social and leisure activities, resulting in more efficient neuronal network functionality which can help delay the clinical manifestations of dementia (Nithianantharajah and Hannan, 2011; Arenaza-Urquijo et al., 2015). This hypothesis is based in the fact that neuroplasticity linked to cognitive experience can construct the necessary reserves for maintenance of brain function, preventing that someone reach the limit of clinical expression of forgetting and loss of other cognitive abilities. In this way, many works show that those strategies can increase brain resilience to future (and sometimes natural) damages (Arenaza-Urquijo et al., 2015; Clare et al., 2017; Wang et al., 2017).

In experimental research, the enriched environment (EE) method is being used to promote cognitive stimulation. There is increasing evidence in the literature showing the benefits of EE on the amplification of synaptic transmission and consequent improvements in cognitive function, a protector factor for memory across lifespan or in the aged phase of life (Redolat and Mesa-Gresa, 2011; Baraldi et al., 2013; Speisman et al., 2013; Sampedro-Piquero and Begega, 2017). Moreover, in transgenic animal models for AD, EE has been shown to be effective in improving memory, although there is still controversy about its effect on the formation and clearance of amyloid plaques, as the effects also depends on the severity of the animal model for the disease (Herring et al., 2011; Beauquis et al., 2013; Montarolo et al., 2013; Hüttenrauch et al., 2017). The aim of this study was to evaluate the density of senile plaques and their clearance in a mouse model of neurodegeneration using EE in the late phase of life (after 8 months of age). It was found that this stimulus was sufficient to disrupt senile plaque formation and was associated with a slight improvement in spatial memory in the transgenic (TG) mice in a model that is similar to late onset AD.

## Materials and Methods

### Animals

The breeding males of hemizygous transgenic mice PDGFB-APPSwInd were acquired from The Jackson Laboratory, Maine, USA (Stock Number 006293) and a colony was established in the animal facility of the Department of Physiological Sciences of Santa Casa de São Paulo School of Medical Sciences, São Paulo, Brazil. These mice express a mutant form of the human amyloid precursor protein. To ensure the presence of the mutant gene, all animals were genotyped following the protocol described by the Jackson Laboratory. Between 2 months and 4 months of age these animals showed structural and functional neuronal loss, even without the formation of senile plaques. At 8–10 months of age 45% of the transgenic mice present Aβ deposits (Hsai et al., 1999). The control group was formed by C57Bl/6 animals (wild type, WT), the genetic control of the transgenic mice. Animals from the same litter were maintained in groups of 3–6 in individually ventilated cages with food and water *ad libitum*. Room temperature was 24°C to 26°C and humidity was 55%.

Every effort was made to reduce the number of animals and their suffering, following the 3 R’s principle (Replacement, Reduction and Refinement). The experimental proceedings were performed according to the “Ethical principles for the use of laboratory animals” described by the Brazilian Society of Laboratory Animal Science (SBCAL, Brazil). The experimental protocols were approved by the Animal Ethics Committee of the Institute of Biomedical Sciences, University of São Paulo (013/99/book 2).

### Enriched Environment

Transgenic (TG-EE, *n* = 6) and WT (WT-EE, *n* = 8) mice were placed in an EE composed by objects that were randomly chosen including ladders, exercise wheels, balls and other objects. At least three objects with different sizes and textures (plastic, glass, wood, stone, fiber) were placed inside each cage. The objects were changed every 2–3 days and the animals were maintained in this environment from 8 months to 12 months of age. Age-matched control groups (TG-Ctrl, *n* = 6 and WT-Ctrl, *n* = 9) were maintained in regular cages and manipulated 2–3 times a week. Before the beginning of experiments, no objects were added to any cage.

### Evaluation of Spatial Memory and Motor Activity

Spatial memory was evaluated using the Barnes maze and the protocol used was based on a previous study (Baraldi et al., 2013). The maze comprised a white arena, 100 cm in diameter positioned 1 m from the floor. The arena had 30 holes (5 cm diameter each), arranged radially. Under one of the holes, there was a dark box (escape box) was placed, filled with bedding material. A black cardboard wall was placed around the arena’s circumference containing four yellow figures to add spatial orientation. Above the center of the arena, a fluorescent lamp was placed. Animals were submitted to the test when they were 12 months old.

On the first day, each mouse was placed in the center of the arena under a round acrylic cover for 1 min. The animal was then released and the time to find the escape box was recorded. The maximum time allowed for the exploration was 5 min. If the animal did not find the correct hole, it was gently conducted to the hole by the experimenter. Once inside the box, the lights were turned off and the animal was left for 5 min. This learning phase was repeated once a day for five consecutive days (the 5th day was considered the “probe day”). One week after the probe day, the animals were again submitted to the maze, and the time to enter the escape box was recorded. The escape box was always placed in the same hole for the same animal but in different holes for different animals. The maze was cleaned using a 5% ethanol solution before the test of each animal.

Motor activity was evaluated using an activity box (model 7430, Ugo Basile, Comerio, Italy) as described previously (Nunes et al., 2015). Briefly, animals were put individually into the apparatus that consisted of a transparent acrylic cage with sets of horizontal and vertical sensors to register the locomotor activity and rearing, respectively. Motor activity was recorded for 5 min.

After the behavioral tests, animals were anesthetized with isoflurane (3%–5%) and killed by decapitation. Brains were removed, immediately frozen in 2-methylbutane (−45°C to −55°C) and stored at −80°C until used. Half of each brain was used to histological analysis, so samples were cut (20 μm) in a cryostat at −22°C to −20°C (Microm HM525, Germany) and sections were mounted on gelatin-coated slides. The other hemisphere was used to biochemical assays. The number of samples used in each analysis is stated below.

### Quantification of Amyloid-β Plaques

Samples of brain tissue from five animals randomly chosen from each experimental group were fixed in 4% paraformaldehyde, washed in distilled water and stained with Congo-Red for 45 min as described previously (Viel et al., 2008; Nunes et al., 2015). The slides were then washed and immersed in alkaline alcohol. They were then immersed in cresyl echt violet for 10 min, followed by washing with alcohol and xylene. Image acquisition and analysis were done using a Nikon Eclipse E600 light microscope (Kanagawa, Japan) connected to a digital system for image acquisition (IP Lab, Scanalytics, MD, USA). Total density of senile plaques, their distribution and the density of neuronal bodies in hippocampus were analyzed according to dorso-ventral division in 12 brain slices.

### Quantification of Amyloid-Beta Oligomers and SR-B1 Densities

Hippocampal tissues from seven WT and six transgenic mice, randomly chosen from each experimental group were homogenized in a lysis buffer containing 50 mM Tris–HCl (pH 7.4), 0.1% Triton X-100, 4 mM ethylene glycol tetraacetic acid (EGTA), 10 mM ethylenediaminetetraacetic acid (EDTA), and a tablet containing protease inhibitors (Complete EDTA-free, Roche Diagnostics, Germany). The supernatant fractions were subsequently centrifuged at 12,000 *g* for 15 min, at 4°C to remove debris and the total protein content was determined using the Bradford protein assay (1976). Proteins (25 μg/lane) were separated by 10% sodium dodecyl sulfate-polyacrylamide gel electrophoresis (SDS-PAGE) and transferred onto PVDF membranes previously incubated with methanol 100% for 5 min. To ensure equal protein loading, the Ponceau method was used to stain the membranes before probing with the antibodies (Salinovich and Montelaro, 1986). Membranes were preincubated with Tris (100 mM, pH 7.6) containing 0.1% Tween-20 and 0.9% saline (TBST) and 5% non-fat milk for 1 h. The membranes were then incubated overnight at 4°C with primary monoclonal mouse anti-amyloid β, clone W0-2 (MABN10, Merck-Millipore, 1:2,000) and scavenger receptor class B member 1 (SR-B1; Novus Biologicals, 1:1,000).

The membranes were washed and then incubated with a secondary antibody, horseradish peroxidase-conjugated Goat-anti-Mouse IgG (ab20043, Abcam), diluted 1:5,000 in TBST for 2 h at room temperature. The immune complexes were detected by chemiluminescence using a digital image system (ImageQuant™ LAS 500, GE, Sweden). After detection, membranes were stripped with stripping buffer (200 mM glycine, 0.1% SDS, 1% Tween-20, pH 2.2) and then incubated with β-tubulin (ab134185, Abcam, 1:6,000) or actin (Sigma-Aldrich, 1:1,000) antibodies. The density of immunoblotting was quantified with ImageJ software (Abràmoff et al., 2004). Data were reported in relation to the intensity of the β-tubulin band.

### Statistical Analysis

Data were expressed as means ± standard error of means (SEM) and analyzed with the Graph Pad Prism program (GraphPad Software, San Diego, CA, USA; version 6). Behavioral data obtained in the Barnes maze were analyzed using two-way ANOVA followed by Bonferroni’s test to compare the effects of stimulation in the EE on spatial memory between the probe day and the test day performed 1 week later. Cellular and molecular data were compared using Student *t*-test (comparison of the presence of senile plaques between the TG control and TG EE groups) or two-way ANOVA followed by Bonferroni’s test. Only *p* values < 0.05 were considered statistically significant.

## Results

### Effect of Enriched Environment on Spatial Memory and Motor Activity

**Figure 1:**
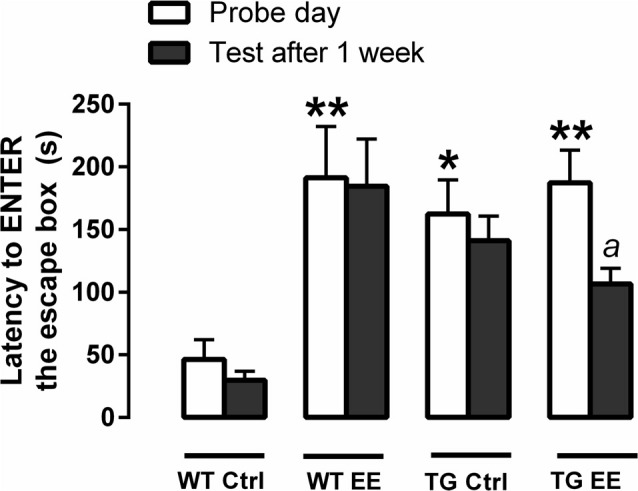
Evaluation of spatial memory of wild type (WT) and transgenic (TG) mice submitted or not to enriched environment (EE) for 4 months. The latencies each animal took to enter the escape box was recorded. Histograms and vertical bars are means ± SEM. *P < 0.05 and **P < 0.01 in relation to the WT-Ctrl group; a: *P* < 0.05 in relation to time spent on the probe day (TG-EE group).

On the probe day of the spatial memory evaluation, it was observed a significant increase in time to enter the escape box in the WT-EE (4.6 times, *P* < 0.01), TG-Ctrl (3.5 times, *P* < 0.05) and TG-EE (4.0 times, *P* < 0.01) groups, when compared to the WT-Ctrl animals (46.5 ± 15.8 s; Figure 1). This indicates that the three groups spent significantly more time exploring the maze before getting inside the escape box.

In respect of long-term memory retention measured 12 week after the probe day, only the TG-EE animals presented a significant decrease (43.2%, 106.5 ± 12.6 s) in the time taken to enter the escape box, when compared to the time recorded in the probe day (187.4 ± 25.9 s). Also, TG-EE showed a non-significant reduction of 24.5% in the time to enter the escape box 1 week after the probe day, when compared to TG-Ctrl, suggesting a slight improve in memory of the trial (Figure 1).

**Figure 2:**
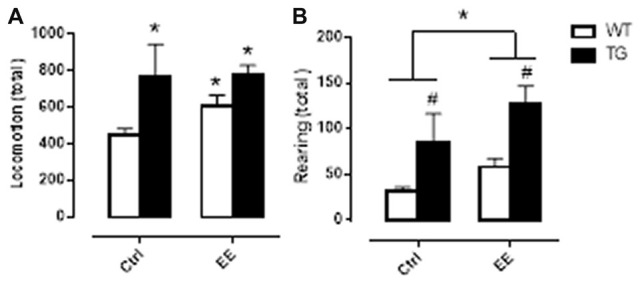
Motor activity evaluation of WT and TG animals, submitted or not to EE. Histograms and vertical bars are means ± SEM. Panel **(A)**: *P < 0.05 in relation to WT Ctrl group. Panel **(B)**: ^#^*P* < 0.05 in relation to WT animals; *P < 0.05.

In order to verify if the greater time observed in the WT-EE, TG-Ctrl and TG-EE groups to enter the escape box on the probe day was related to an increase in motor activity, the animals were submitted to an activity cage immediately after the end of the Barnes maze observations. Corroborating what was observed in Barnes maze, the three groups presented greater locomotion and rearing activities, suggesting that both genetic manipulation and EE stimulation increased the animal’s motor activity (Figure 2).

### Effect of Enriched Environment on Senile Plaque Deposition

**Figure 3:**
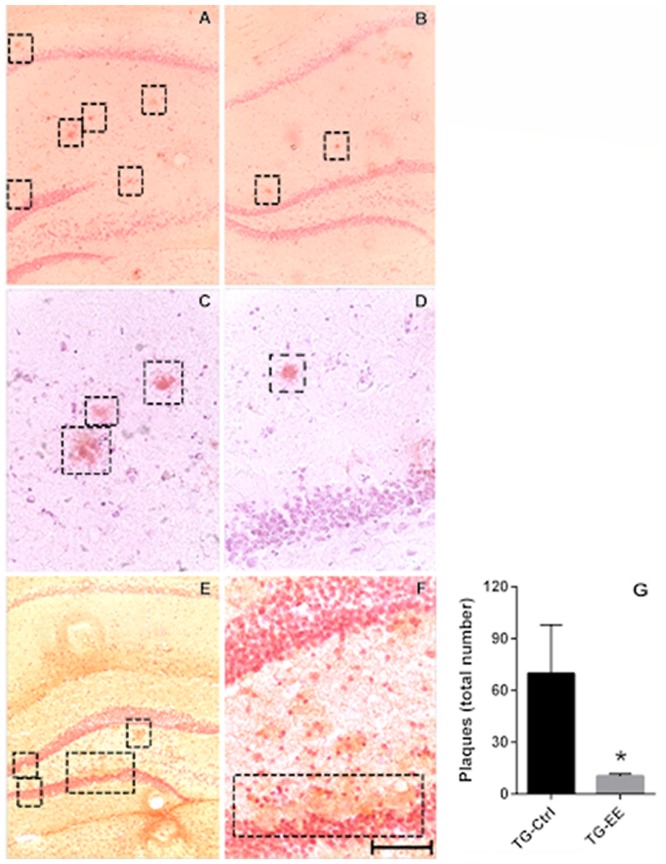
**(A,C)** Photomicrography of the density of senile plaques in TG-Ctrl samples. **(B,D)** Photomicrography of the density of senile plaques in TG-EE samples. **(E,F)** Photomicrography of the dentate gyrus disruption with the presence of senile plaques. Scale bar is: 100 μm to **(A,B,E)** 50 μm to **(C,D,F,G)**. Quantification of senile plaque density in transgenic mice hippocampus (histograms and vertical bars are means ± SEM); *P < 0.05.

The potential of the EE to inhibit the formation of senile plaques was evaluated in stimulated and non-stimulated TG mice. At 12 months of age the TG animals presented 5.49 ± 2.12 senile plaques/total of slices. Submission of TG animals to the EE significantly decreased the density of senile plaques in 69.2% (1.69 ± 0.15 plaques/total of slices, *P* < 0.05), when compared to TG-Ctrl mice (5.49 ± 2.12 plaques/total of slices, Figure 3).

The cortex and amygdala were also analyzed, but no senile plaques were observed in these areas.

**Figure 4:**
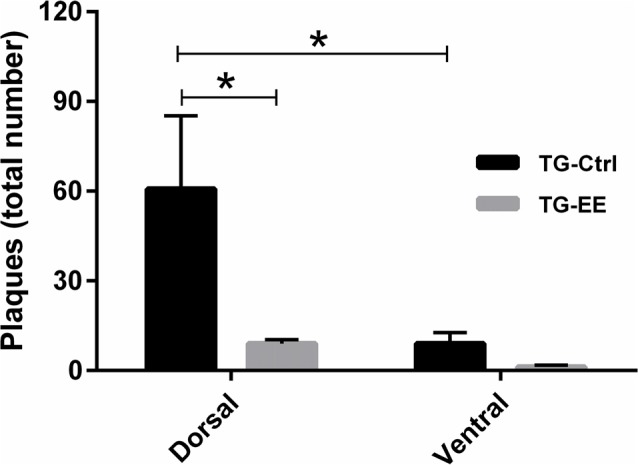
Density of senile plaques in dorsal and ventral portions of the hippocampus of transgenic mice for Alzheimer’s disease (AD) submitted or not to EE. Histograms and vertical bars are means ± SEM. *P < 0.05.

In a comparison of the distribution of senile plaques between the dorsal and ventral regions of the hippocampus in the TG-Ctrl and TG-EE groups, it was found that the concentration of plaques in the dorsal region of the TG-Ctrl group was significantly greater (60.75 ± 24.50 plaques/slice, *P* < 0.05) than in the ventral region (9.00 ± 3.76 plaques/slice). Animals submitted to the EE presented a significant reduction of 85.1% in the density of senile plaques in the dorsal portion of the hippocampus (9.00 ± 1.35 plaques/slice, *P* < 0.05; Figure 4).

### Effects of Enriched Environment on Oligomer Accumulation

**Figure 5:**
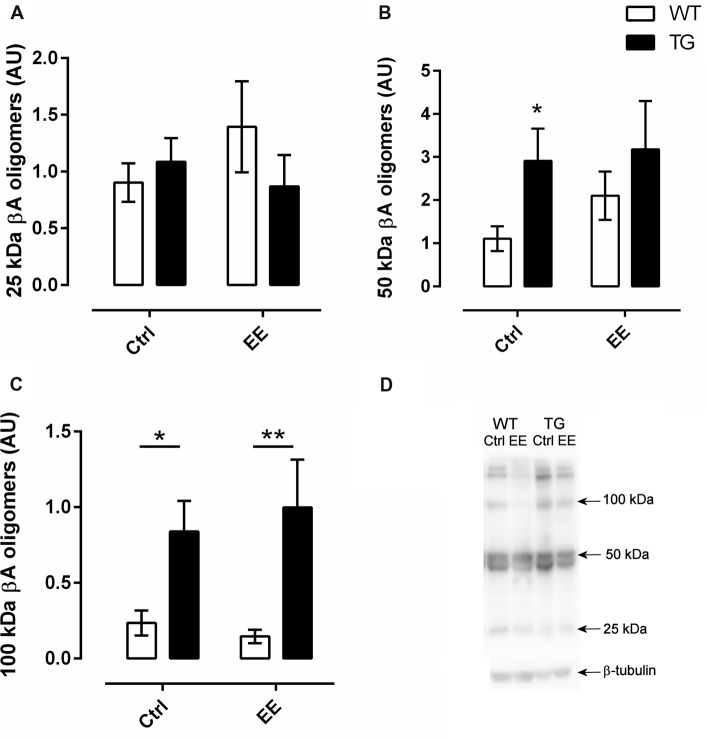
Quantification of amyloid-β (Aβ) oligomers in the hippocampus. Oligomers of three different molecular weights were analyzed. There was no significant difference in relation to 25 and 50 kDa oligomers **(A,B)**. However, transgenic mice showed greater levels of 100 kDa oligomers **(C)**, independently of living in an EE or not. *N* = 5 in all groups. Histograms and vertical bars are means ± SEM. *P < 0.05, **P < 0.01. **(D)**, Illustrative SDS-PAGE results of Ab oligomers from the different groups.

In order to verify if the EE could influence oligomer accumulation, the western-blotting technique was used to detect Aβ oligomers of three different molecular weights. In relation to 25 kDa oligomer, no difference between the strains was observed, with or without the submission of animals to the EE (Figures 5A,D). For the 50 kDa Aβ oligomers, the TG-Ctrl group (2.91 ± 0.75 AU, *P* < 0.05) showed a three-fold increase compared to WT-Ctrl (1.11 ± 0.29 AU; Figures 5B,D). However, the submission of animals to EE did not change the density of these oligomers.

In the same way, there was a significant increase in accumulation of 100 kDa Aβ oligomers in TG mice compared to WT mice (*F*_(1,27)_ = 18.10, *P* < 0.001). This accumulation was not changed in animals submitted to EE (Figures 5C,D).

### Enriched Environment Increased the Clearance of Amyloid-β Plaques

**Figure 6:**
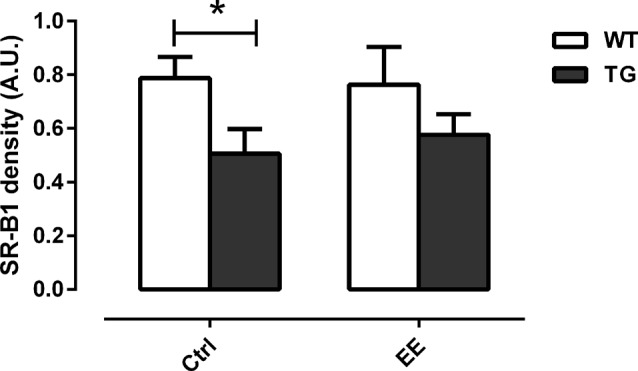
Density of scavenger receptor class B member 1 (SR-B1) in hippocampal homogenates of the different groups. Histograms and vertical bars are means ± SEM. *P < 0.05.

To assess whether the submission of transgenic animals to EE could increase the clearance of Aβ plaques, the density of the SR-B1 was evaluated. Transgenic animals presented a significant decrease of 35.8% in the density of SR-B1 compared to WT animals (0.79 ± 0.08 AU, Figure 6). With the stimulus of EE for 4 months, a slight increase of 1.13-fold in the density of SR-B1 was observed in the TG-EE (0.58 ± 0.08 AU) group compared to TG-Ctrl animals (0.51 ± 0.09 AU).

## Discussion

Among many factors, biological aging increases oxidative stress (Texel and Mattson, 2011; Guillaumet-Adkins et al., 2017), alters energetic homeostasis (Yuan et al., 2016), and increases the accumulation of proteins that cause cellular injuries and lesions in nucleic acids (Maynard et al., 2015). All these changes in the central nervous system decrease the body’s defenses and this may affect learning and memory processes and daily life activities.

The memory loss commonly found in the elderly does not necessarily prejudice daily life. However, this is not the case in the presence of dementias that are associated with some chronic diseases, AD being one of the most frequent of these.

As is widely known, the deposition of Aβ plaques and the intracellular accumulation of Aβ oligomers are some of the pathological features of AD, together with the formation of neurofibrillary tangles. These features can be modified by cognitive stimulation, which alters the structure of the central nervous system of humans and animals, leading to biochemical changes, morphological alterations in synapses and neurons, and increases in neuroplasticity when compared to humans or animals that are not submitted to stimulation (Baraldi et al., 2013; Bonfiglio et al., 2018). Clinical studies show that people, even with only a few years of formal education, can form cognitive reserves and have less chance of developing dementia (Farfel et al., 2013).

In line with these ideas, in the present study an EE was used as cognitive and physical stimuli for transgenic mice that hyper express the human amyloid precursor protein (PDGFB-APPSwInd) and for non-transgenic genetic controls (C57Bl/6), as a strategy to attempt to modify the evolution of the neurodegenerative process.

WT and TG animals were exposed to the EE only when they were 8 months old because, by this age, most of TG animals present the characteristic senile plaques (Hsai et al., 1999). When the animals were 12 months old, they were submitted to a spatial memory evaluation over five consecutive days. On the last day (considered the probe day), the TG-Ctrl, WT-EE and TG-EE animals took longer to enter the escape box and finish the trial compared to WT-Ctrl animals. They spent more time exploring the maze. Previous studies have already shown that transgenic animal models for AD presented greater motor activity because of cortical and hippocampal atrophy (Belzung and Griebel, 2001; Gil-Bea et al., 2007). Furthermore, stimulation of WT and TG animals in an EE also promoted an increase in motor activity. There is no consensus in the literature whether EE stimulation produce increases or decreases in motor activity. Corroborating the behavioral effects observed in the present study, other works also showed similar behaviors leading to improvement in brain activity as well. In this way, it was already shown that EE corrected motor deficits produced by injection of NMDA antagonist MK-801 in pup rats (Nozari et al., 2014). In another work where EE was used previously to induction of brain ischemia, it was observed that this strategy produced brain ischemic tolerance through the increase in physical activity (Xie et al., 2013). The increase in motor activity of rats was also reported after animals were submitted to EE from weaning to adulthood with important and long-lasting antidepressive and anxiolytic effects (Mosaferi et al., 2015). Concerning that we worked with older animals and although the explanation for that motor enhance is not clear in the literature, an increased expression of the protein irisin was observed after resistance exercise in serum and soleus muscle in both aged rats and humans which could prevent age-related decline in muscle function (Kim et al., 2015). However, the relationship of irisin levels and stimulation in EE remains to be elucidated. Moreover, it is fact is that cognitive functioning of old rats is more affected by an impoverished environment (without physical or social activities) than young rats. Similarly, sedentary and lonely people (impoverished environment) have worse cognitive functioning and show a faster cognitive decline than active people (Volkers and Scherder, 2011).

Although the WT-EE and TG-EE groups presented greater motor activity than the WT-Ctrl animals, only TG-EE mice showed significant better performance in respect of long-term spatial memory measured 1 week after the probe day. There is a great deal of literature indicating that an EE improves physiological functions and leads to better cognitive performance in animal models of epilepsy, traumatic brain injury, brain hypoperfusion and also in normal aging (Baraldi et al., 2013; Zhang et al., 2013; Dunkerson et al., 2014; Kotloski and Sutula, 2015). In the present study, the decrease in the time to enter the escape box was indicative of better spatial memory, even though the TG-Ctrl animals did not present significant memory loss.

In this respect and due to the short time of the experimental protocol used in this study, the behavioral observations did not always reflect the molecular benefits of a strategy used to improve quality of life. Corroborating this, although the TG-Ctrl animals did not present a significant loss in spatial memory, there was a significant presence of senile plaques in the hippocampus of the TG-Ctrl mice at 12 months of age. In addition, the submission of the TG mice to an EE for 4 months prevented an increase in this density. A deeper analysis of this distribution showed that the majority of the plaques were concentrated in the dorsal region of the hippocampus, and that this great number was significantly reduced with the stimulus in EE. It is already known that the dorsal region of hippocampus is related to the formation of spatial memory (Fanselow and Dong, 2010; Wang et al., 2012). It is possible that the EE could have promoted a cognitive reserve in these animals, improving their attention and leading to the observed decrease in the latency to find the escape box in the Barnes maze.

Concerning the Aβ oligomers, it is known that small, diffusible prefibrillar amyloid species present great neurotoxic potential than mature fibrillar forms and may affect, in a significant way, different cell signaling pathways (Haass and Selkoe, 2007; Pimplikar, 2009) leading to the cognitive deficits observed with the development of the disease. In this respect, it was observed that animals that presented a reduced quantity of toxic oligomers, presented intact memory function (Lesné et al., 2008). In the present study, the presence of senile plaques and 50 and 100 kDa Aβ oligomers were verified in the hippocampus of transgenic mice, however, no small, low-weight oligomers were detected. The toxicity of the oligomers is controversial in the literature and, apparently, depends on their size and shape. Some important studies can be found which attempt to elucidate the molecular mass and shape of neurotoxic oligomers. Corroborating our findings, studies with 56 kDa and 80 kDa oligomers showed their toxic potential. With the 56 kDa oligomer, a transient, but not permanent memory impairment was reported (Lesné et al., 2006; Larson and Lesné, 2012). In another report, an 80 kDa *in vitro* prepared oligomer solution was similar to the oligomers extracted from an AD human brain (Sebollela et al., 2014). However, in a recent work it was verified that in brain samples from AD patients, high molecular weight (HMW) Aβ oligomers (more than 70 kDa) have little or no cytotoxic activity in many bioassays. These HMW oligomers can be dissociated chemically into low molecular toxic oligomers (8–70 kDa; Yang et al., 2017). In the present work we showed that Tg animals had an increased density of 100 kDa oligomers and significant increased density in 50 kDa oligomers, but this was not observed in stimulated animals. Also, there was no difference in 25 kDa oligomers in all groups. So, the submission of 8 month-old TG-Ctrl animals to an EE for 4 months did not change the quantity of oligomers in the hippocampus, but did change the density of the amyloid plaques. In the same way, a greater time exposure to an EE would probably have resulted in improved memory, as shown before by our research team with old C57Bl/6 mice (Baraldi et al., 2013). The longer the time of stimulation, the better the effects, and this may also have an effect on the quantity of oligomers.

It is important to note that it was also verified that 12 months old C57Bl/6 (Control) animals presented low and HMW oligomers. The presence of Aβ oligomers in old mice was recently reported (Sérrière et al., 2015), but the physiological meaning for this presence is still lacking. In this way, more studies are needed to clarify the physiological role and the toxicity of low molecular and HMW oligomers in old WT mice.

In addition to this, a significant decrease of SR-B1 in the TG-Ctrl animals compared to the WT-Ctrl was observed, unlike in the TG animals submitted to an EE, which showed a slight increase of 1.13-fold in the density of this scavenger receptor. SR-B1 is one of the scavenger receptors that are expressed in microglia mediating their adhesion to fibrillar Aβ and triggering the clearance of Aβ peptides (Paresce et al., 1996; Thanopoulou et al., 2010). Considering that the animals used in this project were older (12 months-old), this suggests that the brains of TG mice that normally have electrophysiological deficits underwent a change after the submission of the animals to the EE, contributing to the built of brain reserve.

In this study, it was demonstrated that keeping transgenic mice for AD in an EE leads to neurophysiological and cognitive alterations, although the time of exposure can determine the behavioral responses found. These physiological changes are numerous and may affect many systems. As cited above, changes in lifestyle may determine the life course and the aging configuration. In this way, positive changes may promote brain neuroplasticity and can contribute to the cognitive reserve. However, these changes may not be experimentally measured, depending on the time point the observation is done. As the organism always tends to a dynamic equilibrium of its functions, stimulation for longer times can lead to a “new physiological state” and the organism adapts and begins to function in this new condition. In this way, the initial structural changes that can be observed and measurable with 4–6 weeks of stimulation, may not be evident after longer stimulation times, as in the present study in relation to the density of neuronal bodies and synaptic terminals (data not shown). Nevertheless, the structural changes as reported here (significant reduction in senile plaques in the dorsal portion of the hippocampus) and the consequent protective alterations are retained and form the brain reserves observed both in humans and animals.

Recent works show that the benefits promoted by environmental enrichment for humans or animals can be studied in molecular levels. This strategy may trigger epigenetic changes that can maintain these long-lasting benefits, although the responsible mechanisms still need further studies. The same can be applied for the benefits of combining pharmacological therapies with environmental enrichment (Sale, 2018). Nevertheless, the data obtained in the present work reveals that EE applied in late-life possibly promoted, at least, brain resistance, once it was observed reduction in senile plaque density and, probably, Aβ clearance were maintained (Arenaza-Urquijo and Vemuri, 2018). As stated earlier, combination of physical and cognitive stimulation that is created in environmental enrichment is clearly linked to preservation of cognitive function (Cheng, 2016), as neuronal structural integrity along with different stimuli (inside the animals cages) can increase motor activity, curiosity and preserve brain function.

In conclusion, strategies such as changing the environment can be used as important complementary therapies to the current pharmacological approaches. These strategies may facilitate the development of brain resilience and resistance that may protect the organism against the neurodegenerative insults. These alterations can alter brain metabolism, reduce neuroinflammation and reduce astrocyte reactivity, for instance, and protect it from the accumulation of amyloid peptides and formation of plaques. The benefits may not be immediate but can be observed in the long-term. Moreover, the findings of the present work, along with those obtained by other groups in human and animal research, point to the real possibility of implementing multimodal programs combining several types of intervention (social interaction, physical and cognitive stimulation, adequate energy intake) to delay the symptoms of AD and improve quality of life in the elderly. The change in lifestyle across lifespan is the key to achieve health span and optimal longevity.

## Data Availability

All datasets generated for this study are included in the manuscript.

## Author Contributions

JB, NS and TV conceived and designed the experiments. JB, NS and GC performed the experiments. NS, HB and TV analyzed the data. TV wrote the article. JB, NS, HB and TV contributed intellectually to the article.

## Conflict of Interest Statement

The authors declare that the research was conducted in the absence of any commercial or financial relationships that could be construed as a potential conflict of interest.
